# Codonopsis pilosula polysaccharides: structural characteristics, structure–activity relationships, biological activities, and potential applications

**DOI:** 10.3389/fchem.2026.1912660

**Published:** 2026-09-03

**Authors:** Bo Wang, Lei Li, Xinyuan Cao, Zhijun Dai, Xiaofeng Liu

**Affiliations:** People’s Hospital of Ningxia Hui Autonomous Region, Ningxia Medical University, Yinchuan, China

**Keywords:** biological activities, *Codonopsis pilosula* polysaccharide, immunomodulation, structural modification, structure-activity relationship

## Abstract

Codonopsis pilosula, a medicinal plant in the Campanulaceae family, is a classic Qi-tonifying herb in traditional Chinese medicine. Research on the pharmacological basis of its activity has spanned several decades. As pivotal active constituents, *Codonopsis pilosula* polysaccharides (CPPs) exhibit strong bioactivities, including immunomodulatory, antioxidant, anti-inflammatory, and antitumor effects. To date, approximately 42 homogeneous polysaccharide fractions have been reported from *C. pilosula* roots, with varying degrees of structural characterization depending on the analytical strategies employed. These CPP fractions exhibit considerable structural diversity in terms of molecular weight, monosaccharide composition, glycosidic linkage patterns, branching architecture, and higher-order conformations. Increasing evidence suggests that these structural characteristics contribute to their diverse biological activities; however, comprehensive understanding of the precise structural determinants underlying CPP bioactivities remains limited. This review systematically summarizes the structural characteristics and pharmacological properties of CPPs, with a specific emphasis on the structure–activity relationships (SAR) underlying their biological effects. We also clarify the molecular mechanisms responsible for their immunomodulatory and antitumor actions and discuss how structural modifications enhance efficacy through well-defined SARs.

## 1 Introduction

*Codonopsis pilosula* (Campanulaceae), a widely used traditional Chinese medicinal herb, has long been applied in traditional Chinese medicine for Qi-tonifying, spleen-invigorating, lung-nourishing, and immune-supporting effects (Gao et al., 2019). Modern phytochemical investigations have identified polysaccharides, saponins, alkaloids, and flavonoids as major bioactive constituents of *C. pilosula*, among which Codonopsis pilosula polysaccharides (CPPs) have attracted increasing attention due to their high abundance and diverse biological activities, including immunomodulatory, antioxidant, anti-inflammatory, metabolic regulatory, and antitumor effects (Bao et al., 2026; Jia et al., 2022; Tang et al., 2022a; Yue et al., 2023). Polysaccharides are structurally complex macromolecules whose biological functions are largely determined by their molecular architectures (Fu et al., 2026). Unlike small-molecule natural products with relatively defined chemical structures, polysaccharides exhibit considerable heterogeneity arising from variations in molecular weight (Mw), monosaccharide composition, glycosidic linkage patterns, branching architecture, and higher-order conformations (Fu et al., 2025). Increasing evidence indicates that these structural parameters collectively determine molecular recognition, receptor interaction, bioavailability, and downstream biological responses, highlighting the importance of establishing structure–activity relationships (SARs) for rational development of polysaccharide-based therapeutics.

During the past decade, several reviews have summarized different aspects of *C. pilosula* and plant-derived polysaccharides (Bai et al., 2025). Previous reviews on CPPs have primarily focused on traditional medicinal applications, extraction and purification technologies, chemical composition, quality control, or general pharmacological activities (Dar et al., 2023; Liang et al., 2024; Luan et al., 2021). Meanwhile, broader reviews of traditional Chinese medicine polysaccharides and plant polysaccharides have mainly discussed common biological properties, particularly immunomodulatory effects, antioxidant activities, and general mechanisms of action across multiple botanical sources (Chen et al., 2022). Although these studies have significantly contributed to the understanding of polysaccharide pharmacology, CPPs have generally been discussed as one representative polysaccharide among many plant-derived materials rather than as a specific molecular system requiring comprehensive structural interpretation (Meng et al., 2023; Zhou et al., 2025a).

Importantly, this review is not intended to provide a simple catalog of pharmacological effects (Pan et al., 2025; Wang et al., 2026b). Instead, it focuses on the chemical and physicochemical basis of CPP function, including molecular weight distribution, monosaccharide composition, glycosidic linkage patterns, branching architecture, charge properties, and higher-order conformation. These parameters are fundamental determinants of polysaccharide solubility, aggregation behavior, receptor recognition, and *in vitro*/*in vivo* bioactivity. Therefore, a structure-centered evaluation of CPPs is essential for understanding their biological effects and for establishing reproducible quality control standards.

Therefore, this review differs from previous reviews in three major aspects. First, instead of providing a general summary of pharmacological activities, we establish a structure-centered analytical framework to clarify how individual structural parameters of CPPs determine specific biological outcomes. Second, we integrate chemical characterization, structure–activity relationship analysis, molecular mechanisms, and structural optimization strategies to explain the transition from structural information to functional effects. Third, we critically discuss current challenges, including structural heterogeneity, insufficient mechanistic validation, inconsistent quality evaluation, and limited clinical translation, and propose future directions for precision development of CPPs-based functional foods, biomaterials, vaccine adjuvants, and pharmaceutical candidates. Through this integrated perspective, the present review aims to provide a more comprehensive understanding of CPPs beyond previous activity-oriented or technology-oriented reviews.

## 2 Structural features of *Codonopsis pilosula* polysaccharides

**FIGURE 1:**
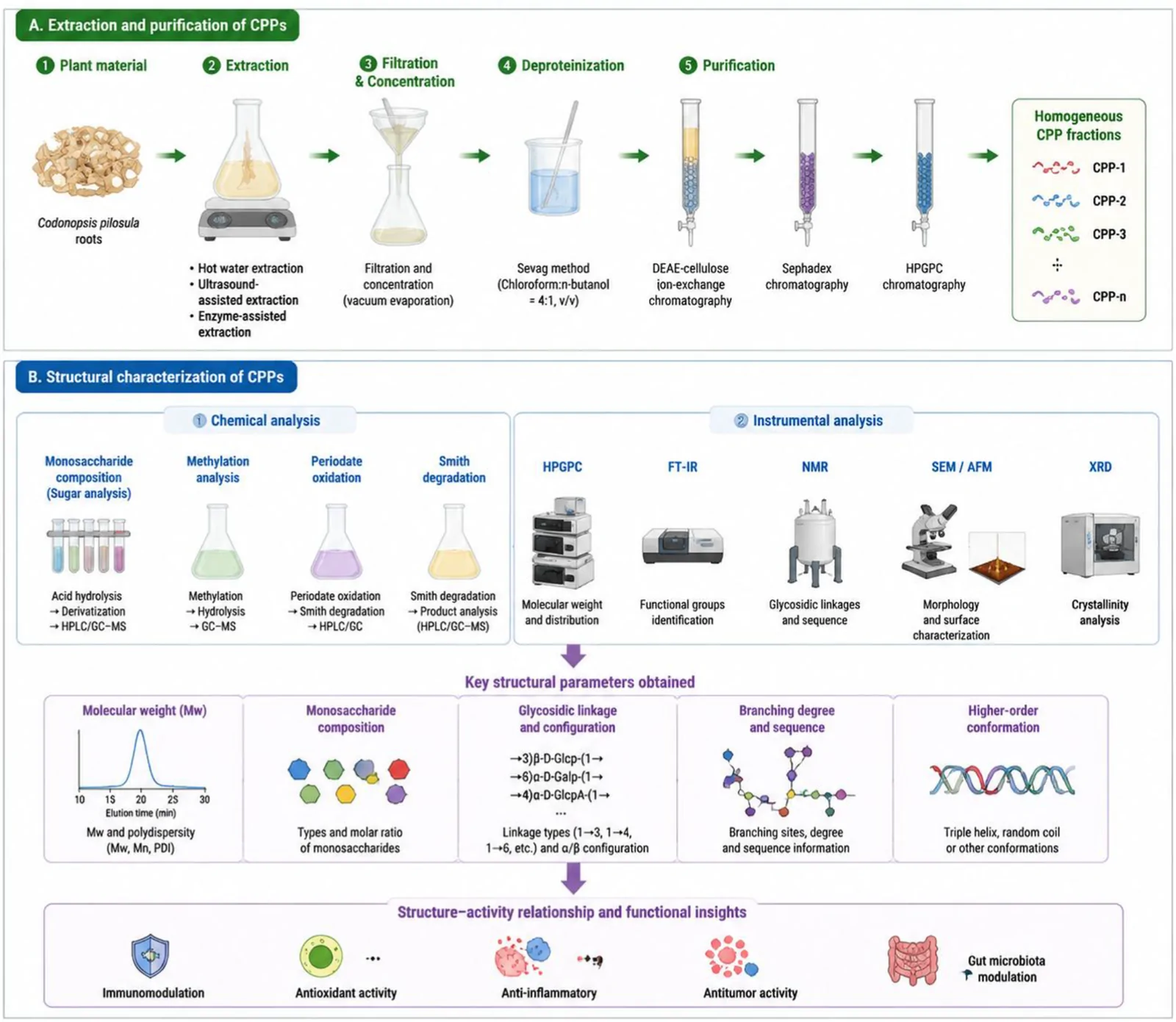
Integrated workflow for extraction, purification, and structural characterization of Codonopsis pilosula polysaccharides. **(A)** Extraction and purification of CPPs; **(B)** Structural characterization of CPPs.

Structural characterization of CPPs relies on an integrated analytical workflow (Figure 1). Molecular weight is commonly determined by HPGPC/HPSEC coupled with MALLS or RI detection, while monosaccharide composition is typically analyzed by GC-MS after derivatization or by HPAEC-PAD. Methylation analysis combined with GC-MS is widely used to determine glycosidic linkage patterns, and NMR spectroscopy provides detailed information on anomeric configuration, residue sequence, and substitution position. In addition, FT-IR, circular dichroism, and microscopic techniques such as AFM or SEM can provide complementary evidence for functional groups, chain conformation, and supramolecular morphology. Because CPPs are highly heterogeneous, no single analytical platform is sufficient; reliable structural assignment requires convergence of multiple techniques. From a physical chemistry perspective, these parameters directly influence solubility, viscosity, chain flexibility, intermolecular association, and conformational stability, thereby shaping biological recognition and activity.

**FIGURE 2:**
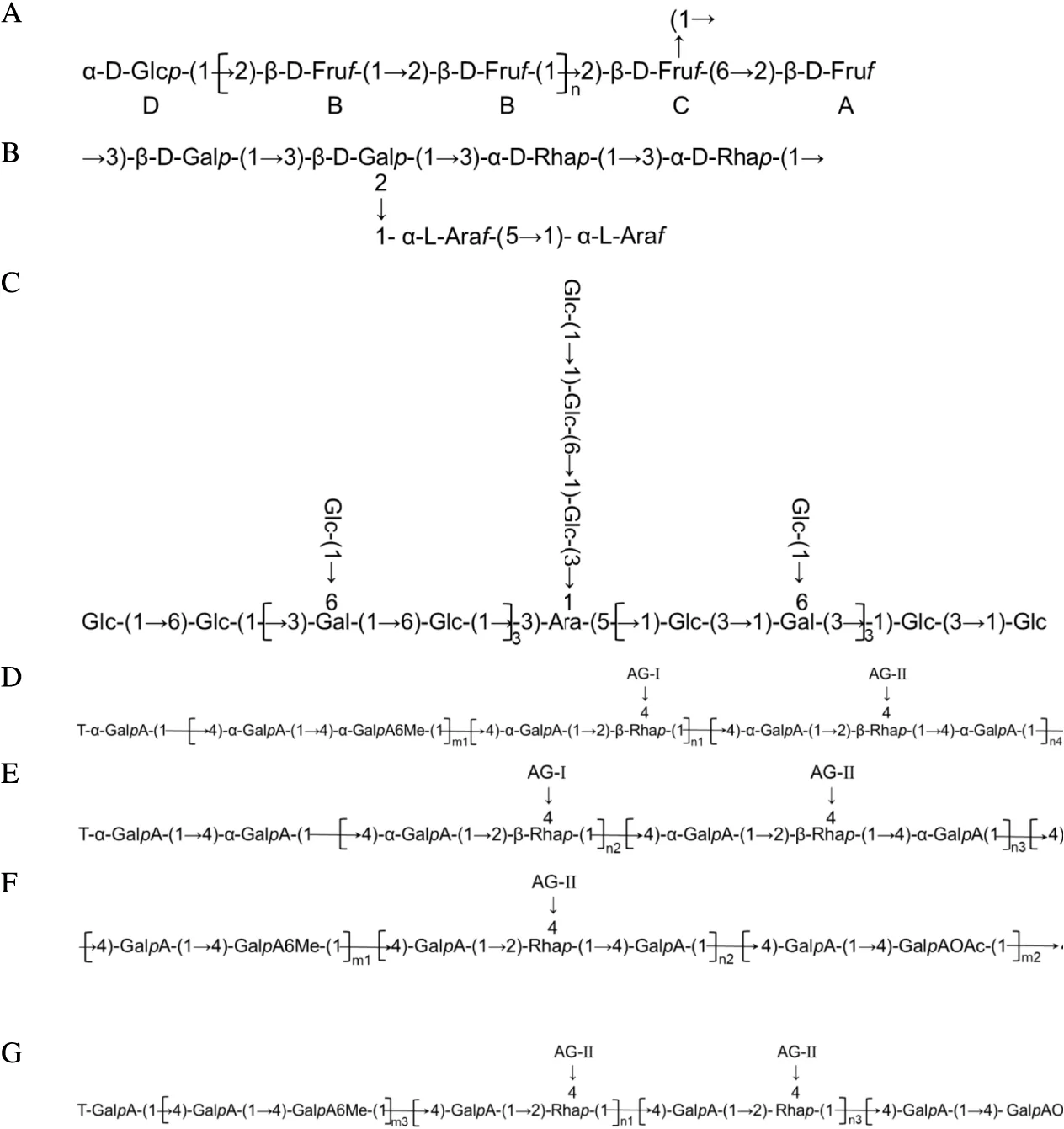
The representative chemical structure of *Codonopsis pilosula* polysaccharides. **(A)** WCP; **(B)** CPP; **(C)** CERP1; **(D)** CPP-1; **(E)** CTP-1; **(F)** CPSP-1; **(G)** CTSP-1.

Structurally, CPPs are classified into two groups: homopolysaccharides, which contain only one type of monosaccharide, and heteropolysaccharides, which comprise two or more different monosaccharides such as glucose, arabinose, galactose, rhamnose, and xylose, with glucose as the predominant component (Wang et al., 2025). It is important to note that extracts from *C. pilosula* yield polysaccharides that are not structurally uniform but represent a mixture of these various structural types (Figure 2). CPPs display a broad molecular weight distribution, ranging from approximately 0.345 kDa–12,900 kDa, with bioactive fractions being chiefly water-soluble. The first water-soluble polysaccharide isolated from *C. pilosula* was shown to possess immunomodulatory properties (Yongxu and Jicheng, 2008). Typical glycosidic bonds identified in CPPs include (1→4)-α-D-Glcp (1→6)-β-D-Galp, and the fructan-type linkages (2→1)-β-D-Fruf and (2→6)-β-D-Fruf. The physicochemical and biological properties of these polysaccharides are closely correlated with specific structural features, including the monosaccharide composition, molecular weight, glycosidic linkage position and configuration, functional groups, branching degree, and higher-order conformation (Fu et al., 2025). A summary of the molecular weight, monosaccharide composition, and key structural characteristics of polysaccharides isolated from *C. pilosula* is provided in Table 1.

**TABLE 1:** Chemical structure and composition of polysaccharides from *Codonopsis pilosula*.

CPPs fraction ID	Molecular weight (kDa)	Monosaccharide composition (molar ratio)	Key structural features	Analytical techniques	Ref
WCP-I	-	GalA:Gal:Rha:Ara:Man:Glc = 69.6:17.6:6.4:5.5:0.7:0.2	β-D-(1→4)-Galactan	GC, GC-MS	Zou et al. (2019)
CPP1c	126	Rha:Ara:Gal:GalA = 3:1:2:33	→1)-α-L-Rhap-(2,4→, →1)-α-L-Araf-(5→, →1)-α-D-Galp-(6→, →1)-α-D-GalpA-(4→	HPGPC, GC, FT-IR, GC-MS, NMR, SEM, TEM	Zhang et al. (2017)
CPPs	4.23	Fru:Glc	(2→1)-β-D-Fruf, (1→)-α-D-Glcp	HPLC, HPGPC, NMR	Ji et al. (2022)
UA-Cpps1	163.8	Ara:Gal:GlcA:Xyl:Rha:Glc:Man = 90.50:4.00:5.75:1.00:2.25:27.5:3.25	Predominantly 1→4 and 1→6 glycosidic bonds	GC, NMR, SEM, AFM	Wang et al. (2025)
UA-Cpps2	0.63	Ara:Gal:Glc:Man = 5.10:1.00:8.00:0.70	Predominantly 1→4 and 1→6 glycosidic bonds	GC, NMR, SEM, AFM	Wang et al. (2025)
UA-Cpps3	0.45	Man:GlcA:Glc:Gal:Ara = 1.22:2.67:7.11:2.19:1	Predominantly 1→4 and 1→6 glycosidic bonds	GC, NMR, SEM, AFM	Wang et al. (2025)
UA-Cpps4	0.67	Man:Glc:Gal:Ara = 1.00:9.13:1.75:4.75	Predominantly 1→4 and 1→6 glycosidic bonds	GC, NMR, SEM, AFM	Wang et al. (2025)
UA-Cpps5	0.55	Man:Rha:Glc:Gal:Ara = 1.13:1.00:17.88:1.38:5.63	Predominantly 1→4 and 1→6 glycosidic bonds	HPLC, GC, NMR, SEM, AFM	Wang et al. (2025)
UA-Cpps6	0.89	Man:Glc:Gal:Ara = 3.00:7.2:1.00:8.00	Predominantly 1→4 and 1→6 glycosidic bonds	GC, NMR, SEM, AFM	Wang et al. (2025)
CERP-1	4.84	Ara:Glc:Gal = 1.00:19.83:6.94	→1,4)-β-D-glucose, →1,3)-β-D-glucose, →1,6)-β-D-glucose, →1,3,6)-β-D-galactose	HPAEC-PAD, HPLC, HPSEC-MALLS, FT-IR, NMR, TEM	Liu et al. (2018)
CPP-1 (Zou et al., 2021)	21	Gal:GalA:Rha:Ara:Glc:Man:Fuc = 12.8:58.9:9.4:16.7:1.1:<0.5:<0.5	HG backbone, RG-I side chains, methyl-esterified GalA	GC, GC-MS, HPSEC-MALS, NMR	Zou et al. (2021)
CTP-1	29.5	Gal:GalA:Rha:Ara:Glc:Man = 11.9:71.0:7.7:8.4:0.8:<0.5	HG backbone, RG-I side chains	GC, GC-MS, HPSEC-MALS, NMR	Zou et al. (2021)
COP-W1	23.4	Man:Rha:Glc:Gal = 20.32:1:1.27:36.13	(1→3)-Gal, (1→3,6)-Man, (1→4)-Man, (1→6)-Man residues	HPGPC, HPLC, FT-IR, GC, NMR	Wu et al. (2020)
PSDSs-1	3.3	Rha:GalA:Glc:Gal:Ara:Fru = 0.05:0.08:1.00:0.13:0.13:1.24	-	HPGPC, HPLC	Li et al., (2022a)
CPP-1 (Ma et al., 2024)	4.89	Fru:Glc:Ara = 29.72:1.00:0.76	α-D-Glcp-(1→[2)-β-D-Fruf-(1→2)-β-D-Fruf-(1]_26_→2)-β-D-Fruf	HPSEC-MALLS-RID, UV, FT-IR, GC-MS, NMR	Ma et al. (2024)
CPPS-I	570	Glc:Man:Gal	α-Type glucan with pyranose ring	GPC, HPLC, FT-IR, NMR, AFM, SEM	Li et al. (2023)
CPPS-II	980	Glc:Man:Gal	α-Type glucan with pyranose ring	GPC, HPLC, FT-IR, NMR, AFM, SEM	Li et al. (2023)
CPPS-III	3,300	Man:Gal:Glc = 2.30:1.62:1	α-Type glucan with pyranose ring	GPC, HPLC, FT-IR, NMR, AFM, SEM	Li et al. (2023)
CPPS	970	Man:Glc:Xyl = 5.8:1.9:1.0	-	HPGPC, HPLC, SEM, DEC	Liu et al. (2023a)
CPW	5.7	Fru:Glc:Gal:Ara = 0.83:0.15:0.07:0.01	-	HPGPC, FT-IR, AFM, SEM	Fu et al. (2025)
CPS0.2	112.9, 6.0 (polydisperse)	Fru:Glc:Ara:Gal = 0.65:0.15:0.12:0.08	-	HPGPC, FT-IR, AFM, SEM	Fu et al. (2025)
CPS0.5	59.6, 24.7, 4.9 (polydisperse)	Fru:Ara:Glc:Gal:Fuc = 0.82:0.09:0.06:0.02:0.01	-	HPGPC, FT-IR, AFM, SEM	Fu et al. (2025)
CPS1	>200, 11.2, 6.5 (polydisperse)	Fru:Ara:Fuc:Glc:Gal:Xyl = 0.61:0.14:0.17:0.04:0.03:0.01	-	HPGPC, FT-IR, AFM, SEM	Fu et al. (2025)
50 WCP-II-I	71.6	Ara:Rha:Fuc:Xyl:Man:Gal:Glc:GlcA:GalA = 5.8:5.7:1.0:1.0:0.4:11.2:2.6:1.3:71.4	HG region (partially methyl-esterified GalA), interrupted by short RG-I region	SEC, GC, NMR	Zou et al. (2014)
100 WCP-II-I	53.2	Ara:Rha:Xyl:Man:Gal:Glc:GlcA:GalA = 7.5:6.4:0.8:0.3:12.4:2.3:1.2:69.0	Two domains: Galacturonan with AG-I side chains on GalA C2; RG-I with AG-II side chains on Rha C4	SEC, GC, NMR	Zou et al. (2014)
CPPs	0.345	Gal:Ara:Glc = 52.2:29.9:13.8	-	HPGPC, HPLC, FT-IR, NMR, SEM	Gao et al. (2020)
RCNP	11.4	Ara:Gal = 75.2:24.8	(1→5)-Araf backbone	HPGPC, GC-MS, NMR	Sun et al. (2019)
RCAP-1	50.9	Rha:Ara:GalA = 5.7:3.5:90.8	Highly methyl-esterified HG backbone	HPGPC, GC-MS, NMR	Sun et al. (2019)
RCAP-2	258	Rha:Ara:GalA = 3.3:3.0:93.7	Highly methyl-esterified HG backbone	HPGPC, GC-MS, NMR	Sun et al. (2019)
CPP1b	145	Rha:Ara:Gal:GalA = 0.25:0.12:0.13:2.51	1,4)-α-D-GalpA, 1,4)-α-D-GalpA6Me, rare 1,2)-β-L-Rhap, 1,2,6)-α-D-Galp	HPGPC, GC-MS, NMR, TEM	Yang et al. (2013)
S-CPPA1	133.2	Glc:Gal:Ara = 10.5:3.4:1.7	(1→4)-Glcp, (1→6)-Galp, non-reducing terminal Glcp	HPGPC, GC, GC-MS	Li et al. (2012)
CP-A	3.6	-	(2→1)-β-D-fructofuranose backbone	HPGPC, NMR	Li et al. (2017)
CPP1-2-1	10.4	-	-	GC-MS, NMR, TEM, SEM	Meng et al. (2020)
CPP1a	101	Rha:Ara:Glc:Gal:GalA = 1.34:12.30:3.49:10.44:1.18	→1)-β-L-Rhap-(4→, →1)-β-Arap-(5→, →1)-β-D-GalpA-(4→, →1)-β-D-Galp-(6→	HPGPC, GC, GC-MS	Bai et al. (2018)
CPPS3	74	Gal:Ara:Rha = 1.13:1.12:1	(1→3)-linked β-GalpNAc, (1→3)-linked α-Rhap, (1→2,3)-β-Galp	GC, HPLC, NMR	Zhang et al. (2010)
CPP	11	Gal:Rha:Ara = 1.12:1.00:1.12	(1→3)-linked β-D-galactopyranosyl, (1→2,3)-linked β-D-galactopyranosyl, (1→3)-linked α-D-rhamnopyranosyl	HPSEC, GC, UV, IR, NMR	Yongxu and Jicheng, (2008)
CPP 2-4	39	Glc	α-1,6-glucan	HPGPC, HPLC, FT-IR, NMR	Lu et al. (2023)
WCP	6.44	Fru:Glc	Fruf-(2→, Glcp-(1→, →1)-Fruf-(2→, →1,6)-Fruf-(2→	HPSEC-MALLS-RI, FT-IR, HPLC, GC-MS, NMR, SEM, X-Ray	Dong et al. (2026)
BCP	15.71	Fru:Glc	Fruf-(2→, Glcp-(1→, →6)-Fruf-(2→, →1)-Fruf-(2→, →1,6)-Fruf-(2→	HPSEC-MALLS-RI, FT-IR, GC-MS, NMR, SEM, X-Ray	Dong et al. (2026)
CPSP-1	13.1	Ara:Rha:Gal:GalA = 8.9:9.3:11.0:70.1	HG + RG-I backbone, AG-II side chains	GC, GC-MS, NMR	Zou et al. (2020)
CTSP-1	23	Ara:Rha:Gal:GalA = 8.2:11.2:18.9:61.3	HG + RG-I backbone, AG-I + AG-II side chains	GC, GC-MS, NMR	Zou et al. (2020)
CP2A	12,900	Gal:Ara = 60.76:39.24	→4)-β-Galp-(1→3,6)-β-Galp-(1→	GPC, HPLC, FT-IR, GC-MS, NMR, HILIC-MS/MS	Zhu et al. (2025)

Abbreviations: GalA = galacturonic acid, GlcA = glucuronic acid, Ara = arabinose, Gal = galactose, Glc = glucose, Rha = rhamnose, Xyl = xylose, Man = mannose, Fuc = fucose, Fru = fructose, HG, homogalacturonan; RG-I, rhamnogalacturonan-I; AG-I, arabinogalactan type I, AG-II, arabinogalactan type II; “-” represents “Not reported”.

## 3 Structure-activity relationship of *Codonopsis pilosula* polysaccharides

**FIGURE 3:**
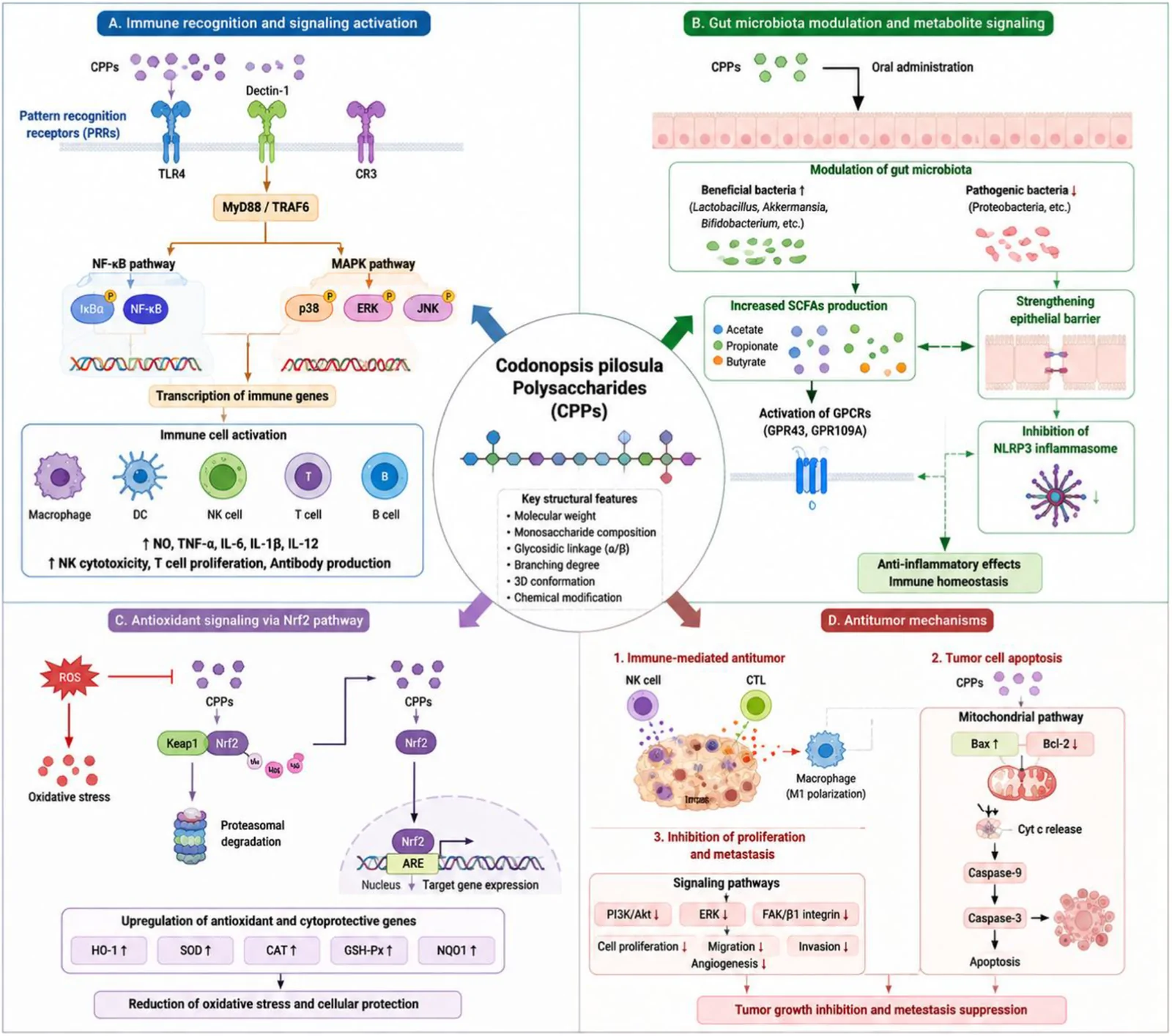
Molecular mechanisms underlying Codonopsis pilosula polysaccharide-mediated biological activities. **(A)** Immune recognition and signaling activation; **(B)** Gut microbiota modulation and metabolite signaling; **(C)** Antioxidant signaling via Nrf2 pathway; **(D)** Antitumor mechanisms.

Although increasing evidence suggests that molecular weight, monosaccharide composition, glycosidic linkage patterns, branching degree, and higher-order conformations contribute to CPP bioactivities, current SAR analyses remain largely correlative rather than fully mechanistically established. The prevailing view is that polysaccharides mediate various bioactivities through distinct mechanisms intrinsically linked to their chemical attributes, including monosaccharide profile, molecular weight, chain conformation, glycosidic linkage type, and structural modifications. Current knowledge regarding the correlation between structural parameters and biological functions of CPPs is summarized in Figure 2, whereas the receptor-mediated molecular mechanisms underlying these structure-dependent activities are illustrated in Figure 3.

### 3.1 Molecular weight and biological activity

Molecular weight (Mw) is one of the most fundamental structural parameters governing polysaccharide function. Polysaccharides obtained from natural sources are generally polydisperse mixtures, and their biological activity depends critically on specific structural details rather than merely the presence of the macromolecule. Variations in molecular weight lead to differences in chain length, flexibility, three-dimensional conformation, and receptor-interaction patterns, which in turn produce distinct biological activities and mechanisms of action (Li et al., 2020). CPPs exhibit a wide molecular weight distribution, from low (e.g., 3.3 kDa) to ultra-high (>2000 kDa), with size being a major determinant of both the nature and intensity of their bioactivity.

Immunomodulatory properties are strongly influenced by CPPs molecular weight. Two CPPs fractions of contrasting molecular weight—PSDSs-1 (3.3 kDa) and PSDSs-2 (>2000 kDa) exerted opposing immunomodulatory effects *in vitro* and *in vivo* (Li et al., 2022a). Specifically, PSDSs-1 increased levels of the pro-inflammatory cytokines TNF-α and IL-6 while decreasing the anti-inflammatory cytokine IL-10. In contrast, PSDSs-2 induced the opposite cytokine profile. This suggests that molecular weight can govern the direction of immune modulation. A positive correlation between immunomodulatory potency and molecular weight is commonly observed for polysaccharides within specific size ranges, often attributed to more defined spatial architectures and multiple receptor-binding epitopes in high-Mw molecules. Nevertheless, this correlation is not absolute. For instance, Ji et al. (Ji et al., 2022) isolated a low-molecular-weight glucan (average Mw ∼4.23 kDa) showing pronounced immunostimulatory activity, indicating that bioactivity is not exclusive to high-Mw fractions.

Similarly, the anti-tumor effect of CPPs is markedly influenced by Mw. Among three membrane-separated fractions—CPPS-I (<60 kDa), CPPS-II (60–100 kDa), and CPPS-III (>100 kDa)—the medium-molecular-weight CPPS-II showed the strongest *in vitro* inhibition of tumor cell proliferation, with high-dose efficacy comparable to that of the chemotherapeutic agent doxorubicin (Li et al., 2023). *In vivo*, co-administration of CPPS-II with doxorubicin resulted in synergistic tumor suppression and enhanced immune function, whereas CPPS-I and CPPS-III were less effective. These findings demonstrate that the anti-tumor activity of CPPs is optimized within a specific molecular weight range.

Antioxidant activity constitutes a major pharmacological basis of CPPs. The correlation between antioxidant activity and Mw is complex and modulated by additional structural parameters. A prevalent view (Chen et al., 2019; Guo et al., 2014; Rotrekl et al., 2021) holds that low-MW polysaccharides generally exhibit stronger antioxidant activity, likely because their structures are more flexible, exposing more active groups such as hydroxyl groups to neutralize free radicals including DPPH, ABTS, and hydroxyl radicals. For instance, Zou et al. (Zou et al., 2020) reported that low-Mw CPPs CPSP-1 and CTSP-1 (13.1 and 23.0 kDa) displayed antioxidant activity via modulation of the intestinal cellular antioxidant defense system. Exceptions to this trend, however, are noted. Sun et al. (Sun et al., 2014) observed that for polysaccharides derived from marine *Chrysophyta*, antioxidant activity increased with higher molecular weight. Additionally, Xu et al. (Xu et al., 2015) suggested that a very low molecular weight could destabilize advanced polysaccharide structures (e.g., the triple helix), potentially diminishing bioactivity. Therefore, the influence of molecular weight on CPPs antioxidant activity likely follows a bell-shaped curve, implying the existence of an optimal molecular weight window.

CPPs display a broad molecular weight distribution, and their bioactivity generally follows a non-linear relationship. In many cases, fractions with moderate molecular weight show stronger biological effects than extremely high- or low-Mw fractions, suggesting an optimal size window for receptor recognition, solubility, and cellular uptake. Excessively large polysaccharides may suffer from poor diffusion and limited bioavailability, whereas overly small fragments may lose the conformational features required for effective bioactivity. Thus, molecular weight should be considered a primary SAR determinant rather than a simple physicochemical descriptor.

### 3.2 Monosaccharide composition and biological activity

CPPs are mainly composed of glucose (Glc), arabinose (Ara), rhamnose (Rha), galactose (Gal), xylose (Xyl), mannose (Man), and galacturonic acid (GalA). Among these, GalA-rich fractions are frequently associated with stronger antioxidant and antitumor activities, likely because acidic groups increase negative charge and may enhance interactions with proteins or cell-surface components. In contrast, Glc-rich fractions, especially those containing β-linked backbones, are more commonly associated with immunomodulatory activity. Therefore, monosaccharide composition is not only descriptive but also predictive of functional bias. β-Glucans are critically important for immune cell activation; clinically used polysaccharide immunoadjuvants, including lentinan (Zhou et al., 2009), certain mushroom polysaccharides (Zavadinack et al., 2025), and Euglena gracilis polysaccharides (Guo et al., 2025), are β-glucans. As shown in Table 1, glucose is a nearly ubiquitous component in CPPs, suggesting that its prevalence contributes significantly to their bioactivity, positioning monosaccharide composition as a key factor in the SAR.

Acidic sugars such as GalA confer a negative charge to the polysaccharide backbone, which can promote stronger interactions with cellular receptors. A substantial body of evidence indicates that plant polysaccharides with strong immunomodulatory activity are often rich in uronic acids. For CPPs, higher GalA content correlates with stronger anti-tumor activity (Bai et al., 2018). Furthermore, electrophilic groups, such as keto or aldehyde groups can facilitate hydrogen release under certain conditions, a mechanism that contributes to antioxidant activity. Li et al. (Li et al., 2022b) isolated two antioxidant polysaccharides, CLRP-1 and CLSP-1, and found that CLSP-1, with a higher proportion of GalA, exhibited more potent bioactivity. Therefore, the content of acidic sugars in CPPs is a significant determinant for enhancing their biological activity.

### 3.3 Effect of glycosidic linkage type

Glycosidic linkage type plays a pivotal role in defining polysaccharide structure and function. Variations in linkage types endow polysaccharides with different physicochemical characteristics and three-dimensional conformations, which in turn modulate their bioactivity (Cheng et al., 2021; Zhu et al., 2024). For example, Wang et al. (Wang et al., 2025) reported that polysaccharides UA-Cpps3 and UA-Cpps5, obtained using ultrasound-assisted extraction and primarily composed of 1→4 and 1→6 linkages, exhibited strong antioxidant and enzyme inhibitory activities. In another study, In another study, two pectic polysaccharides (CPSP-1 and CTSP-1) showed favorable antioxidant activity (Zou et al., 2020). Structural analysis revealed that the side chains of CTSP-1 consisted of both arabinogalactan type I (AG-I) and type II (AG-II), whereas CPSP-1 possessed only AG-II, which was speculated to be a major factor contributing to CPSP-1’s superior performance in reducing oxidative stress markers. Glycosidic linkage patterns are also crucial for immunomodulatory activity. RCAP-1 and RCAP-2 from *Codonopsis pilosula* roots (Sun et al., 2019), which have backbones mainly composed of long, highly methyl-esterified homogalacturonan (HG) regions with α-(1→4)-linked GalA, significantly promoted nitric oxide production in macrophages. In contrast, RCNP, which had a backbone of (1→5)-linked Araf and (1→4)- (1→6)-, or (1→3)-linked Galp residues, did not. The activity difference between RCAP-1 and RCAP-2 was further related to the length of the HG region and the degree of methyl-esterification (DM), suggesting that a higher DM may enhance immunostimulatory activity.

Beyond neutral and acidic polysaccharides, specific linkages also define the activity of other types. An inulin-type fructan CP-A with a backbone of (2→1)-linked β-D-fructofuranose exerted significant anti-ulcer effects in an ethanol-induced gastric ulcer model, dose-dependently increasing antioxidant enzyme activities (SOD, GSH-Px) and decreasing ulcer index and inflammatory markers (MDA, NO, MPO) in gastric tissue (Li et al., 2017).

Glycosidic linkage type strongly affects polysaccharide conformation and receptor recognition. β-(1→3)- and β-(1→6)-linked structures, particularly when combined with appropriate branching, are generally more potent in immune activation, as they are more likely to be recognized by pattern-recognition receptor systems such as TLR4-and Dectin-1-associated pathways. In contrast, certain arabinogalactan-type or pectic structures may contribute more prominently to antioxidant or anti-inflammatory effects depending on their overall architecture. Branching is usually beneficial when it maintains structural accessibility, but excessive branching may reduce receptor engagement.

### 3.4 Effects of processing methods on structure and activity

Processing methods such as steaming, roasting, stir-frying, and honey-processing can alter polysaccharide composition, molecular weight, and branching features, thereby affecting bioactivity. These changes may improve extraction efficiency, enhance solubility, or expose functional groups, but they may also degrade native structural motifs that are required for activity. Therefore, processing should be viewed as an important structural regulator rather than a simple preparative step. Exposure to high temperatures, pressure, and adjuvants such as honey, rice, or clay during processing unavoidably induces polysaccharide degradation, oxidation, Maillard reactions, and other structural alterations (Wu et al., 2018; Xue et al., 2018). The most direct effects of processing on CPPs are observed in their extraction efficiency and chemical makeup. Heat treatment generally breaks down plant cell walls to facilitate the release of intracellular components. Tang et al. (Tang et al., 2022b) evaluated the *in vitro* antioxidant activity of four processed clover products and found that honey processed clover had a higher content of oligosaccharides than raw products, exhibiting stronger activity. This may be due to the introduction of sugar from honey. However, fructose and glucose from honey may also interfere with monosaccharide composition analysis of CPPs. High temperatures during stir-frying can cause cleavage of glycosidic bonds, leading to thermal degradation. Rice-stir-fried *C. pilosula* (Zou et al., 2017) shows significantly reduced CPPs content and markedly increased 5-hydroxymethylfurfural (5-HMF), which stimulates gastrointestinal smooth muscle contraction *in vitro* and may contribute to the enhanced spleen-invigorating effect of processed products. Mechanistically, this may be related to synergistic regulation of N2 receptors and β-adrenergic receptors on gastrointestinal smooth muscle. Processing can also affect polysaccharide solubility and morphology. High-temperature treatment may increase cell wall permeability, leading to polysaccharide release or degradation (Gu et al., 2022), while physical friction during processes such as rice-stir-frying may alter the tissue microstructure, affecting extraction and activity. Another common method, bran-frying, uses wheat bran as an adjuvant to enhance the spleen-invigorating effect. Wang et al. (Wang et al., 2018) showed that bran-frying reduces the content of both CPPs and volatile components, likely due to high-temperature degradation or interaction with components in the wheat bran. However, there is currently limited research on the specific effects of bran-frying on the fine structure of CPPs, warranting further investigation.

### 3.5 Impact of structural modification on activity

Chemical modification is an effective strategy to optimize the bioactivity of CPPs. Common derivatization approaches include sulfation, phosphorylation, carboxymethylation, and selenylation (Liu et al., 2025a; Liu et al., 2025b; Tong et al., 2026; Yang et al., 2024). These modifications can alter charge density, hydrophilicity, molecular conformation, and receptor-binding behavior, thereby modulating biological functions. In general, moderate modification often enhances activity, whereas excessive substitution may disrupt the native conformation and reduce functional performance. Therefore, the biological outcome of modification depends not only on the modification type, but also on the degree of substitution and the preservation of the active backbone.

Sulfation is one of the most widely used modification strategies. The introduction of sulfate groups increases the negative charge density and water solubility of CPPs, which may improve interactions with immune-related receptors and strengthen antioxidant, antiviral, and immunomodulatory activities. Sulfated CPPs derivatives have been reported to show stronger antioxidant and hepatoprotective effects than native polysaccharides (Liu et al., 2015a). Similarly, sulfation can yield new or potentiated biological effects, as demonstrated by the synergistic action of sulfated CPPs and polygonatum polysaccharide against Newcastle disease virus (Liu et al., 2015b). Phosphorylation represents another key approach. Ming et al. (Ming et al., 2017) reported that phosphorylated CPPs surpassed its native counterpart in suppressing Duck Hepatitis A Virus virulence, providing direct evidence that modification can potentiate specific biological functions.

Phosphorylation is another important derivatization method. By introducing phosphate groups, phosphorylation can improve the physicochemical properties of CPPs and promote their biological recognition. Phosphorylated CPPs have been shown to exhibit enhanced antiviral and immunoregulatory activities compared with their unmodified counterparts, suggesting that phosphate substitution may strengthen the interaction between polysaccharides and biological targets (Ming et al., 2017).

Carboxymethylation mainly improves water solubility and can change the spatial conformation of polysaccharide chains, thereby increasing accessibility to biological targets (Chakka and Zhou, 2020). This modification is often associated with improved antioxidant and immunomodulatory activities (Hu et al., 2024; Liu et al., 2023b). Although fewer studies have focused specifically on carboxymethylated CPPs, this strategy is considered promising for enhancing functional properties while maintaining a polysaccharide-based backbone.

Selenylation introduces selenium into the polysaccharide structure and may confer additional redox and immunoregulatory properties. Qin et al. (Qin et al., 2016) found that selenylated CPPs significantly enhanced lymphocyte proliferation and elevated the CD4+/CD8+ T-cell ratio, indicating potentiated immunostimulatory activity. In addition, In addition, CPP-based nanoformulations, such as CPP-SeNPs, further expand the application potential of modified CPPs by integrating polysaccharide bioactivity with nanomaterial-mediated delivery advantages (Hu et al., 2022; Long et al., 2024).

Overall, structural modification provides a feasible strategy for improving the functional performance of CPPs. However, the relationship between modification type, substitution degree, conformational preservation, and biological outcome remains insufficiently understood. Future studies should combine rigorous structural characterization with bioactivity evaluation to establish more precise structure–function relationships for modified CPPs.

## 4 Biological activities of *Codonopsis pilosula* polysaccharides

Accumulating evidence indicates that CPPs exert diverse biological activities through a hierarchical mechanism involving structural recognition, receptor activation, intracellular signaling regulation, and microbiota-mediated systemic responses (Figure 3). However, these activities should not be interpreted as independent pharmacological events, because they are largely interconnected through common biological processes involving pattern-recognition receptor activation, oxidative stress regulation, inflammatory signaling, immune cell remodeling, and microbiota-mediated metabolic regulation (Table 2).

**TABLE 2:** The biological activities of the polysaccharides purified from Codonopsis pilosula (Increase, ↑; Decrease, ↓).

Compound name	Types (*In vitro* or *In vivo*)	Experimental model	Dosage	Effect	Key signaling pathways	Ref
WCP-Ia	*In vitro* and *In vivo*	Peyer’s patch cells; C3H/HeJ mice (female)	*In vitro*: 10 and 20 μg/mL; *In vivo*: 100 mg/kg	IL-6↑, TGF-β↑, TNF-α↑, sIgA↑, CD4+/CD8^+^ T lymphocyte↑	Not explicitly reported	Zou et al. (2019)
CS-GO-CPP	*In vitro*	RAW264.7 cell	0.78, 1.56, 3.13, 6.25, 12.5 μg/mL	NO↑, IL-4↑, IFN-γ↑, CD40↑, CD86↑, CD 88↑	NF-κB pathway	Sun et al. (2022)
CPP1c	*In vitro* and *In vivo*	*In vitro*: mouse splenocytes; *In vivo*: Male SAMP8 mice	*In vitro*: 50, 100 and 200 μg/mL; *In vivo*: 200 mg/kg	splenocytes: percentage of CD4^+^, CD8^+^, CD28^+^ and CD152+ T cells↑; IL-2↑, TNF-α↑, IFN-γ↑, CD28, PI3K and p38MAPK mRNA↑	TCR/CD28 signaling pathway	Zhang et al. (2017)
CPPS	*In vivo*	Male BALB/c mice	100 mg/kg	TLR 4↓, Foxp 3↓, CD 4 + T↑, IL-2↑, IL-2α↑	Not explicitly reported	Zheng et al. (2014)
sCPPS	*In vivo*	Female ICR mice	0.05, 0.1, 0.15 mg/mL	IgG↑, IgM↑, IFN-γ↑, IL-2↑, IL-4 ↑	Not explicitly reported	Gao et al. (2020)
CPP	*In vivo*	BALB/c mice	50, 100, 200 mg/kg	IFN-γ↑, IL-2↑, IL-10↑, IgG↑	Not explicitly reported	Fu et al. (2018)
RCAP-1	*In vitro*	RAW264.7	40 μg/mL	NO↑	Not explicitly reported	Sun et al. (2019)
CPP-SeNPs	*In vivo*	SPF-grade Kunming mice	0.75, 1.5 mg/kg	TNF-α↑, IFN-γ↑, and IL-2↑, NK cell cytotoxicity↑, macrophage phagocytosis capacity↑	Mitochondrial apoptosis pathway (Bax↑, Bcl-2↓)	Long et al., 2024)
CPCP	*In vivo*	Female C57BL/6 mice	714 mg/kg	CD68+macrophage count ↓, IL-1↑, IL-6↑, iNOS↑, TNF-a↑	Not explicitly reported	Liu et al. (2021)
CPPA	*In vitro*	HO-8910 cells	25, 50, 100, and 200 g/mL	CD44 expression↓	Not explicitly reported	Xin et al. (2012)
CPAP	*In vivo*	Female Kunming mice	50, 100 mg/kg	Lactobacillaceae ↑, *Alloprevotella* contents↓, T cell↑	Not explicitly reported	Fan et al. (2025)
CPP	*In vivo*	mice	100 mg/kg	cleaved caspase-1↑, NLRP3↑, ASC↑, GSDMD↑, IL-1β↑, IL-18↑	NF-κB signaling pathway	Huang et al. (2024)
CPW1-Se	*In vitro*	HepG2 and Huh-7 cells	12.5, 25, 50 μg/mL	Cleaved Caspase-9↑, Cleaved Caspase-3↑, Cycle A2 and Bcl-2 protein expression↓	Mitochondrial apoptosis pathway	Hu et al. (2022)
CPP1-2-1	*In vivo*	Male C57BL/6 mice	100 mg/kg	TLR4↓, NF-κB↓, TNF-α↓, IL-6↓	TLR4/NF-κB signaling pathway	Meng et al. (2020)
CPPs	*In vivo*	mice	300,600, 1,200 mg/kg	g__*Ligilactobacillus*↑, g_*Akkermansia*↑, g_*Faecalibaculum*↑, g_*Odoribacter*↑, acetic acid↑, butyric acid↑, SCFA↑, NLRP3↓	SCFA/GPR/NLRP3 signaling pathway	Zhou et al. (2025a)
CPP	*In vivo*	Mice	300, 200, 100 mg/kg	TC↓, TG↓, LDL-C↓, ALT↓, AST↓, MDA↓, IL-6↓, and TNF-α↓, HDL-C↑, SOD↑, and GSH-Px↑	AMPK/ACC/SREBP1 signaling pathway	Chen et al. (2025)
CPP-A-1	*In vivo*	Mice	50, 100 mg/kg	ALT↓, AST↓, α-SMA↓, ECM↓, MDA↓, iNOS↓, SOD↑, GSH↑, Mn-SOD↑, TNF-α↑, IL-6↑, IL-11	TLR4/NF-κB, TGF-β1/Smad3 signaling pathways	Meng et al. (2023)
COP-W1	*In vitro*	-	0.15, 0.3, 0.6, 1.2, 2.4, and 4.8 mg/mL	DPPH↑	Not applicable	Wu et al. (2020)
SCP	*In vitro*	RAW264.7 cells	​	SOD↑, GDH-Px↑, CAT↑, NO↑, MDA↓, ROS↓, iNOS↓	Not applicable	Yang et al. (2021)
CPPS	*In vitro*	liver homogenate	0.1, 0.5, 1, 2, 4, 8 mg mL	lipid peroxidation↓	Not applicable	Liu et al. (2023a)
UA-Cpps	*In vitro*	-	-	DPPH↑, ABTS^+^↑, OH^−^↑, Reducing Power↑	Not applicable	Wang et al. (2025)
CP	*In vitro* and *In vivo*	*In vivo*: Female ICR mice	*In vitro*: 0.010, 0.020, 0.039, 0.078, 0.156, 0.313, 0.625, 1.25, 2.5 and 5 mg/mL; *In vivo*: 100, 150, 200 mg/kg	DPPH↑, ABTS^+^↑, OH^−^↑; SOD↑, GSH-Px↑, MDA↓	Not applicable	Liu et al. (2015a)
CERP1	*In vivo*	male C57BL/6J mice	150,300,600 mg/kg	TC↓, TG↓, LDL↓, NEFA↓, LDL/HDL ratio↓, T-AOC↑, SOD↑, CAT↑, GSH-Px↑, ALT↓, AST↓, AKP↓	Not applicable	Liu et al. (2018)
S-CPPA1	*In vivo*	Mice	10 mg/kg	BUN↑, Cr↑, TNF-α↑, LDH↑, AST↑	Not applicable injury	Li et al. (2012)
POL	*In vivo*	Male ICR mice	0.25, 0.5 and 1.0 g kg	LG↑, MG↑, BUN↓, LDH↓, MDA↓, GSH↑	Not applicable	Xie et al. (2020)
CP-A	*In vivo*	Sprague Dawley rats	50 mg/kg	SOD↑, GSH-Px↑, MDA↓, NO↓	Not applicable	Li et al. (2017)

### 4.1 Immunomodulatory effects

Immunomodulation represents the most extensively investigated activity of CPPs. Current evidence suggests that CPPs regulate immunity through two complementary mechanisms: direct activation of immune cells (Li et al., 2023; Liu et al., 2021)and indirect regulation through the intestinal microbiota (Li et al., 2022a).

Among immune cells, macrophages are primary targets of CPPs. Several CPPs fractions, including GalA-rich pectic polysaccharides and fructan-type polysaccharides, stimulate macrophage activation by enhancing phagocytosis, increasing CD86/MHC-II expression, and promoting cytokine production (Feng et al., 2025; Ji et al., 2022). Mechanistically, these effects are mainly associated with activation of TLR4-mediated MyD88/NF-κB and MAPK signaling pathways (Gao et al., 2025; Zheng et al., 2014). However, structural dependence is evident because different CPPs fractions exhibit distinct immune profiles. For example, highly methyl-esterified homogalacturonan-rich fractions RCAP-1 and RCAP-2 strongly promoted NO production in RAW264.7 macrophages, whereas arabinogalactan-rich fractions showed weaker activity, suggesting that uronic acid content and backbone architecture may influence receptor recognition (Sun et al., 2019). CPPs also regulate adaptive immunity by promoting T-cell proliferation and cytokine secretion. Pectic polysaccharide WCP-Ia increased CD4+/CD8+ T-cell ratios and enhanced IL-6, TNF-α, and TGF-β production in immunosuppressed mice (Zou et al., 2019). Similarly, selenylated CPPs derivatives exhibited enhanced immunostimulatory activity through stronger NF-κB activation, indicating that structural modification can improve immune potency (Qin et al., 2016).

Beyond direct immune-cell activation, CPPs regulate systemic immunity through the gut microbiota. In cyclophosphamide-induced immunosuppressed mice, CPPs administration restored immune organ indexes, increased immunoglobulin production, improved intestinal barrier integrity, and enriched beneficial bacteria associated with short-chain fatty acid production (Fu et al., 2018; Xie et al., 2025). These findings suggest that CPPs-mediated immunoregulation represents a multi-level process involving immune receptors, intestinal barrier repair, microbial remodeling, and metabolite signaling.

Nevertheless, current evidence remains mainly preclinical. Although TLR4 and microbiota-dependent mechanisms have been proposed, direct receptor-binding studies and well-defined structure–function models remain insufficient. Future studies should combine glycomics, receptor screening, and immune profiling to identify precise immunoactive structural motifs.

### 4.2 Anti-tumor activity

Malignant tumors remain a major global public health problem. Despite significant advances in treatments such as surgery, radiotherapy, and chemotherapy, issues persist including severe side effects, drug resistance, and limited efficacy (Ying and Hao, 2023). CPPs exhibit antitumor activity through multiple complementary mechanisms, including direct tumor cell killing, immune activation, inhibition of metastasis, and enhancement of chemotherapy efficacy. Certain CPPs fractions directly induce tumor cell death. For example, CPPs activated NLRP3 inflammasome-mediated pyroptosis in non-small-cell lung cancer cells, resulting in GSDMD cleavage and release of IL-1β and IL-18 (Huang et al., 2024). Other fractions induce mitochondrial apoptosis through regulation of Bax/Bcl-2 and caspase pathways. CPPs also suppress tumor progression by enhancing host immunity. CPPs-modified selenium nanoparticles increased TNF-α, IFN-γ, and IL-2 levels, enhanced NK-cell cytotoxicity and macrophage phagocytosis, and inhibited H22 tumor growth in mice (Long et al., 2024). Furthermore, CPPs can inhibit tumor metastasis. Liu et al. (Liu et al., 2017) found that a *C. pilosula* polysaccharide (CLPS), exhibited potent activity against melanoma metastasis. In a B16F10 lung metastasis model, CLPS administration decreased pulmonary metastatic nodules. *In vitro* analysis indicated that CLPS inhibited melanoma cell migration by blocking β1 integrin binding, suppressing focal adhesion assembly, and downregulating the β1 integrin/FAK/paxillin signaling pathway.

Moreover, CPPs can influence tumor microenvironment remodeling. Medium molecular weight CPPS-II exhibited stronger antitumor activity than lower or higher molecular weight fractions, indicating that optimal molecular size may be required for effective immune interaction and bioavailability (Li et al., 2023). Remarkably, CPPs can also act synergistically with conventional chemotherapy. In a mouse model of colitis-associated cancer, Zhou et al. (Zhou et al., 2025b) found that co-administration of a *C. pilosula* inulin-type fructan (CP-A) with 5-fluorouracil (5-FU) suppressed tumorigenesis more effectively than 5-FU alone. The combination therapy reduced pro-inflammatory cytokines, suppressed the EGFR/AKT/ERK pathway in tumors, and favorably remodeled the gut microbiome by enriching beneficial genera like *Lactobacillus* and increasing colonic levels of short-chain fatty acids. This study demonstrates that CPPs can enhance chemotherapy efficacy by modulating both oncogenic signaling and the tumor microenvironment. Despite promising findings, antitumor evidence remains limited to cell and animal studies. Clinical validation, pharmacokinetic characterization, and standardized preparation of active CPPs fractions remain major challenges.

### 4.3 Anti-inflammatory activity

The anti-inflammatory effects of CPPs are closely related to their immunomodulatory properties, particularly regulation of macrophage activation, inflammatory cytokine production, and inflammasome activity (Li et al., 2017). In DSS-induced colitis models, CPPs fractions reduced disease severity by inhibiting macrophage infiltration and suppressing TLR4/NF-κB signaling (Meng et al., 2020). In addition, microbiota-dependent mechanisms have become increasingly recognized. CPPS treatment increased SCFA-producing bacteria and enhanced acetate and butyrate production, which activated GPR43/GPR109A signaling and subsequently suppressed NLRP3 inflammasome activation (Zhou et al., 2025a). Furthermore, in naturally aged mice, oral administration of a CPPs fraction (CPP-1) attenuated multi-organ inflammation, an effect closely linked to modulation of the gut microbiome and the gut-liver axis (Zou et al., 2023).

Beyond the gut, CPPs also alleviate inflammation in metabolic and hepatic diseases. In a mouse model of non-alcoholic fatty liver disease (NAFLD), CPPs reduce lipid accumulation and inflammation by modulating the AMPK/ACC/SREBP1 signaling pathway (Chen et al., 2025). CPPs also protect against inflammation-associated metabolic disorders. In hepatic fibrosis models, CPPs simultaneously regulated TLR4/NF-κB and TGF-β1/Smad3 pathways, indicating that CPPs may suppress inflammation through coordinated regulation of immune signaling and tissue remodeling (Meng et al., 2023).

Research has also identified CPPs effects in more complex inflammatory scenarios and through novel molecular targets. For instance, CPPs alleviate sepsis in a model by modulating regulatory T cells (Zheng et al., 2014), and CPPs-based microcapsules exhibit antibacterial and anti-inflammatory effects in wound healing (Dong et al., 2024). Mechanistic studies have revealed diverse targets, such as specific microRNAs and proteins in the context of placental inflammation, reflecting the multifaceted nature of CPPs anti-inflammatory actions (Sun et al., 2025). Regarding safety, numerous preclinical studies suggest CPPs are well-tolerated. An anti-tumor fraction (CPPS-II) showed a good safety profile *in vivo* and alleviated chemotherapy-induced toxicity (Li et al., 2023), and an ibuprofen-CP delivery system demonstrated potential to reduce kidney injury associated with classic NSAIDs (Xu et al., 2022).

Although multiple inflammatory models support the efficacy of CPPs, most studies have focused on crude polysaccharide fractions. The precise structural determinants responsible for anti-inflammatory activity remain unclear, and standardized bioactive fractions are required for future translational development.

### 4.4 Anti-oxidant activity

Oxidative stress, characterized by an imbalance between the production of reactive oxygen/nitrogen species (ROS/RNS) and the body’s antioxidant defenses, is a key contributor to the pathogenesis of numerous diseases, including cardiovascular, neurodegenerative, and metabolic disorders (Liu et al., 2025c). CPPs-mediated antioxidant activity involves both direct chemical scavenging and activation of endogenous antioxidant systems. For example, CPPs have shown scavenging capabilities against DPPH, ABTS, hydroxyl, and superoxide anion radicals (Wang et al., 2025). This intrinsic activity can be further enhanced by structural modification, as evidenced by sulfated CPPs derivatives displaying improved ABTS radical scavenging ability (Liu et al., 2015a). Similarly, fermentation processing can yield CPPs fractions with high antioxidant activity, capable of chelating metal ions and eliminating various radicals (Yuan et al., 2020). Beyond chemical antioxidant assays, CPPs activate intracellular defense pathways. In oxidative-stress-induced intestinal epithelial cells, CPP-1 and CTP-1 reduced ROS accumulation and lipid peroxidation through activation of Nrf2 signaling (Zou et al., 2021). In a neuronal model, CP treatment attenuated Aβ1-40-induced ROS elevation and mitochondrial impairment in PC12 cells. This cytoprotective effect was linked to the downregulation of CD38 expression and the restoration of intracellular NAD + levels, highlighting a mechanism involving cellular energy metabolism (Hu et al., 2021). Animal studies further demonstrated that CPPs increased SOD, CAT, and GSH-Px activities while decreasing MDA levels (Ma et al., 2024). Furthermore, in *Codonopsis elegans*, CPPs treatment (CLRP-1, CLSP-1) dose-dependently lowered ROS and MDA levels while enhancing overall antioxidant status. Mechanistic investigation indicated that these polysaccharides promoted the nuclear translocation of the key longevity and stress-resistance transcription factor DAF-16 (Li et al., 2022b).

However, antioxidant activity evaluated by chemical assays does not necessarily predict biological efficacy. Therefore, future research should emphasize cellular and *in vivo* validation and clarify the relationship between specific structural motifs and antioxidant signaling pathways.

### 4.5 Hypoglycemic activity

Diabetes mellitus is a metabolic disorder characterized by chronic hyperglycemia, with a rising global prevalence that makes it a serious public health concern (Wang et al., 2026a). The search for safe and effective glucose-lowering agents is an ongoing priority. *Codonopsis pilosula*, recognized as a classic “medicinal food” herb, has demonstrated promise in this regard, with modern studies verifying its hypoglycemic activity. Research indicates that CPPs can effectively modulate blood glucose and associated metabolic parameters. Fu et al. (Fu et al., 2008) reported that oral administration of CPPs for 1 week at doses of 100–300 mg/kg/day effectively lowered fasting blood glucose and serum insulin levels while increasing SOD activity in mice. Further supporting this, Liu et al. (Liu et al., 2018) found that a purified polysaccharide, CERP1, significantly decreased fasting blood glucose, improved oral glucose tolerance, and reduced insulin resistance in diabetic model mice. The underlying hypoglycemic mechanism of CERP1 involved a combination of effects, including elevating antioxidant enzyme activities (SOD, CAT, GSH-Px), reducing oxidative stress markers (MDA), promoting hepatic glucose metabolism, and improving lipid profiles.

### 4.6 Other biological activities

Beyond the primary activities discussed, CPPs exhibit a range of other bioactivities, including facilitation of cellular repair, anti-fatigue properties, and Reno protective effects. For instance, CPPs demonstrate significant organ-protective properties. Li and his colleagues (Li et al., 2012) isolated a homogeneous polysaccharide (S-CPPA1, Mw 133.2 kDa) from *Codonopsis pilosulastems*, composed of glucose, galactose, and arabinose (molar ratio 10.5:3.4:1.7) and featuring a branched structure with linkages including (1→4)-Glcp and (1→6)-Galp. The study was the first to report that S-CPPA1 could protect against renal ischemia/reperfusion injury in rats, likely via suppression of the pro-inflammatory cytokine TNF-α. Oral S-CPPA1 (10 mg/kg/day) significantly attenuated the injury-induced elevations in serum urea nitrogen, creatinine, TNF-α, lactate dehydrogenase, and alanine aminotransferase, and ameliorated renal tissue pathology in the model. An acute toxicity study indicated no apparent toxicity at oral doses up to 1,000 mg/kg. Similarly, in the context of gastric protection, Li et al. (Li et al., 2017) reported that CP-A, an inulin-type fructan with a (2→1)-linked β-D-fructofuranose backbone isolated from C. pilosularoots, showed gastroprotective effects in an ethanol-induced rat model of acute gastric ulcer. CP-A treatment (50 mg/kg) significantly enhanced gastric tissue SOD and GSH-Px activities, reduced MDA and NO levels and MPO activity, and lowered the gastric mucosal ulcer index. Furthermore, CPPs possess anti-fatigue activity. Xie et al. (Xie et al., 2020) evaluated the anti-fatigue activity of a Codonopsis polysaccharide in ICR mice. POL significantly prolonged loaded swimming exhaustion time, increased post-exercise hepatic and muscle glycogen stores, decreased serum blood urea nitrogen, lactate dehydrogenase, and malondialdehyde, and raised glutathione levels. In conclusion, the broad spectrum of pharmacological activities identified for CPPs through extensive research underscores their significant potential to beneficially influence human health across multiple physiological systems.

Although CPPs have been reported to exhibit immunomodulatory, antioxidant, anti-inflammatory, antitumor, and other bioactivities, the current evidence base remains heterogeneous and sometimes difficult to compare across studies. Reported discrepancies are likely attributable to differences in botanical origin, extraction and purification procedures, molecular weight distribution, monosaccharide composition, branching structure, impurity profiles, and experimental models. In addition, many studies rely on crude or partially purified fractions, making it difficult to assign bioactivity to a single structural determinant. Therefore, the apparent diversity of CPP activities should be interpreted cautiously, and more standardized, head-to-head comparisons using well-defined fractions are needed to determine whether distinct structures preferentially drive specific biological outcomes.

## 5 Systematic evaluation of the current research status of *Codonopsis pilosula* polysaccharides

### 5.1 Development and commercial applications of *Codonopsis pilosula* has polysaccharide

As a traditional medicinal herb, *Codonopsis pilosula* has been used for centuries in Asia, and its long history of use provides ethnopharmacological relevance rather than clinical proof of efficacy. Currently, a considerable number of commercial products containing *C. pilosula* are available. According to publicly available market information, 125 approved medicinal products listing *C. pilosula* as an ingredient are marketed, including granules and oral solutions (National Medical Products Administration, 2006). In addition, 73 C. pilosula products are registered as health foods in China, with claims related to immune enhancement, fatigue alleviation, and gastric protection (National Institute of Metrology, 2026). However, most of these products are formulated from crude extracts or whole-herb preparations rather than purified CPPs, indicating that CPPs-specific product development remains limited.

As the major macromolecular bioactive constituents of *C. pilosula*, CPPs have attracted increasing attention from the food and pharmaceutical industries. This interest is reflected in the growing number of patents related to CPPs (Table 3), which mainly demonstrate development potential and technical exploration in pharmaceuticals, functional foods, cosmetics, and animal nutrition. However, patents and commercial interest should not be interpreted as evidence of validated clinical efficacy. At present, the available evidence for CPPs is still predominantly preclinical, derived mainly from *in vitro* experiments and animal models. Clinical evidence remains limited, and well-designed human studies are still needed to establish safety, efficacy, optimal indications, and practical dosing regimens. Therefore, although CPPs show considerable translational promise, their development into clinically established products remains at an early stage.

**TABLE 3:** List of patents for products containing *Codonopsis pilosula* polysaccharides and their claimed beneficial effects on improving human health.

No	Application	Main composition	Pharmacological properties	Publish number	Product stage	Patent status
1	Animal Science	*Codonopsis pilosula* polysaccharide, *Poria cocos* polysaccharide, Fucoidan	Anti-viral	CN119700803A	Claimed formulation only; no pre-clinical/clinical validation reported	Application published
2	Pharmaceutical	*Codonopsis pilosula* polysaccharide	Immunomodulation	CN118697778A	Claimed formulation only; no pre-clinical/clinical validation reported	Application published
3	Pharmaceutical	*Codonopsis pilosula* polysaccharide	Anti-inflammatory	CN117285658A	Claimed formulation only; no pre-clinical/clinical validation reported	Application published
4	Health product	*Codonopsis pilosula* polysaccharide, Tea polyphenol extract	Anti-oxidant	CN117179094A	Claimed formulation only; no clinical validation reported	Application published
5	Skincare products	*Codonopsis pilosula* polysaccharide, *Lilium* polysaccharides, *Angelica sinensis* polysaccharide	Anti-skin aging	CN115887287A	Claimed formulation only; no clinical validation reported	Application published
6	Animal Science	*Codonopsis pilosula* polysaccharide	Extend the storage time of semen	CN115624024A	Claimed formulation only; no pre-clinical validation reported	Application published
7	Pharmaceutical	*Codonopsis pilosula* polysaccharide, *Glycyrrhiza* polysaccharide	Gastroprotective activity	CN115286721A	Claimed formulation only; no pre-clinical/clinical validation reported	Application published
8	Functional materials	*Codonopsis pilosula* polysaccharide	Anti-bacterial activity	CN115010958A	Claimed formulation only; no performance validation reported	Application published

### 5.2 Quality control and standardization of *Codonopsis pilosula* polysaccharides

Because CPPs are heterogeneous macromolecules, their reported structures and bioactivities are highly sensitive to multiple pre-analytical and analytical variables. First, the botanical source is a primary determinant of composition (Sheng Meng et al., 2023). Differences in species, cultivation conditions, geographical origin, soil, climate, and harvest time can alter polysaccharide yield, molecular weight distribution, monosaccharide composition, and branching patterns (Li et al., 2022b). In addition, postharvest processing, including drying, storage, and traditional processing methods, may induce depolymerization, conformational changes, or partial degradation, thereby affecting both structural characterization and bioactivity evaluation (Liang et al., 2024; Wang et al., 2023). Second, extraction conditions strongly influence the chemical profile of CPPs. Hot-water extraction, ultrasound-assisted extraction, microwave-assisted extraction, enzyme-assisted extraction, and subcritical-water extraction often yield fractions with different molecular weights, solubility, and monosaccharide ratios (Cao et al., 2021; Wu et al., 2021). Harsh conditions may promote hydrolysis or structural rearrangement, whereas mild conditions may preserve native conformations but reduce extraction efficiency. Therefore, extraction protocols should be clearly reported and optimized according to the intended structural and functional endpoints. Third, purification procedures are essential for reproducibility. Crude CPPs preparations often contain proteins, polyphenols, pigments, salts, and possible endotoxin contamination, all of which can confound activity interpretation (Jin et al., 2021). Sequential deproteinization, decolorization, dialysis, ion-exchange chromatography, and gel-permeation chromatography can improve purity (Chen et al., 2016; Shi, 2016), but over-processing may also remove minor active components or alter the apparent activity profile. Thus, purity should be defined not only by carbohydrate content but also by the levels of protein, phenolics, nucleic acid, residual solvent, and endotoxin. Fourth, analytical methodology has a substantial impact on structural assignment. Molecular weight determined by HPGPC/HPSEC-MALLS, monosaccharide composition determined by GC-MS or HPAEC-PAD, linkage analysis by methylation-GC-MS, and conformational information obtained from NMR, FT-IR, and microscopic techniques should be integrated rather than interpreted in isolation (Chen et al., 2024; Ferreira et al., 2015). Because different analytical platforms may produce non-comparable values, standardized workflows and reference materials are needed to improve cross-study consistency.

In future studies, minimum reporting standards for CPPs should include botanical origin, harvest time, extraction conditions, purification workflow, yield, purity, molecular weight distribution, monosaccharide composition, linkage pattern, and contamination profile. Establishing such quality control criteria will facilitate structure–activity comparisons, improve reproducibility, and accelerate the development of CPPs as functional foods or therapeutic candidates. Beyond structural and mechanistic studies, rigorous quality control and standardization are urgently needed to ensure reproducibility and meaningful comparison across studies.

## 6 Conclusions and future perspectives

Research on CPPs, the principal bioactive constituents of this traditional herb, has advanced significantly. This review has summarized their structural characteristics, pharmacological activities, and the current understanding of structure–activity relationships. CPPs constitute a structurally diverse class of biomacromolecules with reported immunomodulatory, antioxidant, anti-inflammatory, antitumor, and hypoglycemic activities. Existing studies suggest that bioactivity is associated with multiple structural factors, including molecular weight, monosaccharide composition, glycosidic linkage types, branching patterns, and chemical modifications.

However, several challenges continue to limit mechanistic understanding and clinical translation. CPPs are highly heterogeneous and polydisperse, which complicates the isolation of homogeneous fractions and weakens cross-study comparability. Most studies still focus on primary structure, while information on higher-order conformation and direct molecular targets remains limited. In addition, many reported findings are based on crude or partially purified fractions, and the contribution of co-existing impurities cannot always be excluded. Although chemical modification can improve activity in some cases, it may also increase heterogeneity and reduce reproducibility if not carefully controlled.

Future studies should prioritize standardized reporting and quality control, including botanical origin, harvesting conditions, extraction methods, purification procedures, purity, molecular weight distribution, monosaccharide composition, linkage analysis, and contamination profiles. Equally important, more rigorous head-to-head comparisons among fractions with controlled structural differences are needed to resolve conflicting findings and establish structure-dependent bioactivity patterns. Mechanistic studies should move beyond pathway association toward direct target identification using receptor-blocking assays, affinity-based approaches, molecular docking with experimental validation, and multi-omics integration. Finally, translational research should expand from cell and animal models to well-designed clinical studies in order to determine safety, efficacy, and real-world applicability of CPPs.

Overall, CPP research is promising but still constrained by structural heterogeneity, inconsistent experimental design, and limited mechanistic validation. Addressing these limitations will be essential for transforming CPPs from empirically active natural products into well-defined bioactive materials with reproducible functions.
